# Retention in Community Health Screening among Taiwanese Adults: A 9-Year Prospective Cohort Study

**DOI:** 10.3390/ijerph19116813

**Published:** 2022-06-02

**Authors:** Huan-Cheng Chang, Ting-Huan Chang, Hsiao-Yen Kang, Yu-Wei Chen, Sheng-Pyng Chen, Mei-Chin Wang, Jersey Liang

**Affiliations:** 1 Division of Family Medicine, Department of Community Medicine, Landseed International Hospital, Taoyuan 324609, Taiwan; changhc@landseed.com.tw (H.-C.C.); kuanghy@landseed.com.tw (H.-Y.K.); chensp@landseed.com.tw (S.-P.C.); 2 Department of Medical Education, Research and Quality Management, Landseed International Hospital, Taoyuan 324609, Taiwan; changtih@landseed.com.tw; 3 Department of Neurology, Landseed International Hospital, Taoyuan 324609, Taiwan; chenyw@landseed.com.tw; 4 Community Health Development Center, Department of Community Medicine, Landseed International Hospital, Taoyuan 324609, Taiwan; wangmeria0406@gmail.com; 5 Department of Health Management and Policy, School of Public Health, University of Michigan, Ann Arbor, MI 48109-2029, USA

**Keywords:** retention, health screening, community, social determinants, cohort study

## Abstract

Largely conducted in Western developed nations, research on community health screening has mainly been of limited duration. This study aims to ascertain the predictors of retention in a community health screening program, involving multiple admission cohorts over a 9-year period in Taiwan. Retention is defined as the participation in subsequent waves of health screening after being recruited for an initial screening. Data came from a prospective cohort study, named “Landseed Integrated Outreaching Neighborhood Screening (LIONS)”, in Taiwan. This research retrieved 5901 community-dwelling Taiwanese adults aged 30 and over from LIONS and examined their retention in three follow-ups during 2006–2014. Generalized estimating equations were employed to evaluate retention over time as a function of social determinants, health behaviors, and health conditions. Being middle-aged, higher education, and regular exercise were positively associated with retention. Conversely, smoking, betel-nut chewing, psychiatric disorder, hypertension, type 2 diabetes mellitus, stroke, and a longer time interval since enrollment were negatively associated with retention. Furthermore, retention rates varied substantially across admission cohorts with more recent cohorts having a lower rate of retention (aOR = 0.33–0.83). Greater attention needs to be directed to retention over time and variations across admission cohorts. Additionally, those who are in either younger or older age groups and have chronic diseases or unhealthy behaviors should be targeted with greater efforts.

## 1. Introduction

Chronic diseases are now the leading causes of mortality and disability, accounting for 70–75% of all deaths globally as well as half of the disability-adjusted life-years lost [1,2]. Moreover, economic losses from noncommunicable diseases have been estimated to reach USD 7 trillion in low- and middle-income countries and exceed USD 47 trillion globally [2,3]. Effective control of chronic diseases is hence a high priority [4,5].

Despite the recognition of the importance of preventive services, their utilization is still suboptimal globally. For example, only 24–60% of Americans were screened for colorectal cancer and 10–34% of adult women received mammography [6]. The partaking rates of the National Health Service (NHS) Health Check in the United Kingdom were around 50% in 2012 [7]. In Singapore, only 39.6% of women aged 50–69 years received mammography, and 47.9% of women aged 25–69 years had a pap smear [8]. Similarly, the proportions of recommended Taiwanese women receiving mammography or pap smears were 39.5% and 56%, respectively, in 2015 [9]. Therefore, much research has focused on the barriers to utilizing preventive services [10,11], and the development of effective preventive care models, including the integration of clinical and community preventive care [12].

Although the predictors of participation in health screening are well documented, there are at least two gaps in the current literature. First, the vast majority of health screening studies have been conducted in Western developed countries [10,12], while little is known about health screening behaviors in Asian countries [8,11]. Because demographic, epidemiological, and economic situations differ substantially across nations [13], their linkages with participation in health screening may vary as well [14,15]. Consequently, replications across a wide range of societies and cultures are crucial to extending the generalizability of research findings based on Western developed nations. Equally important, useful lessons could be drawn from such research for public health programs not only in low- and middle-income nations but also in high-income countries.

Second, prior research mainly consisted of cross-sectional or two-wave short-term longitudinal studies, but multi-wave longitudinal research over an extended period of time has been rare [16]. Given that limited duration and intensity are a major cause of the modest impacts of community-based prevention trials, some investigators have called for longer-term interventions beyond 2 or 3 years [17]. In practice, health screening programs may entail multiple follow-ups of several cohorts with different intervals over an extended period of time because of resource and logistic constraints. Compared to the participation in health screening, little is known about the retention of participants over time [18], which would be important in estimating and interpreting the performance and impact of community-based interventions.

To fill these gaps, this research aims to analyze the retention of adults who participated in a multiple-wave community-based health screening program in Taiwan between 2006 and 2014. Taiwan offers an ideal context to analyze participation in community health screening, where mortality due to major chronic diseases increased from 57.6% in 1981 to 65.5% in 2016 [19]. The burden of chronic diseases is further exacerbated by rapid population aging with the elderly constituting 40.6% of the population in 2060 [20]. Consequently, the integrated health screening for chronic diseases in communities has been a public health priority [21]. Moreover, Taiwan differs substantially from Western developed countries in its culture, health system, and lifestyle. For instance, older Taiwanese, particularly women, are less educated and more likely to live with their children [13]. Furthermore, the national health insurance in Taiwan has led to excellent access to health services, including certain preventive care (e.g., health screening). Further, the prevalence of health risk factors in Taiwan has not only increased significantly (e.g., obesity) [22], but also distributed disproportionately (i.e., smoking and betel-nut chewing), especially prevailing in less-educated men [23,24].

To analyze retention over time, we have identified three groups of influential factors from the literature, including social determinants (i.e., age, gender, education), health behaviors (i.e., smoking, drinking, betel-nut chewing, regular exercise), and health conditions (e.g., psychiatric disorder, hypertension, diabetes). Furthermore, we have applied the Andersen’s behavioral model of health services utilization to assess the impacts of population characteristics (i.e., predisposing, enabling, and need factors) on individuals’ retention behavior in community health screening services [25]. The Andersen’s framework has been employed in numerous studies to examine the predictors of critical health screening services [26,27,28,29]. Theoretically, predisposing (i.e., age, gender, education) and enabling (e.g., psychological/psychiatric status) factors could have both direct and indirect effects on the need and use of health screening services. Moreover, need factors are the most immediate cause and mutable determinants of health screening use. Specifically, perceived need (e.g., the implications of smoking, drinking, betel-nut chewing, and regular exercise) can help to understand the seeking and adherence to health screening services, but evaluated need (e.g., hypertension, diabetes, stroke) will be more related to the utilization of medical care treatment.

We are particularly interested in evaluating the following hypotheses for which there are limited knowledge and mixed evidence. First, prior Western studies indicate that older and younger adults have lower rates of participation in health screening [15]. However, this age-related curvilinear relationship has not been fully established in non-Western countries. Thus, we aim to evaluate this hypothesis of the curvilinear relationship between age and retention in health screening in Taiwan.

Second, betel-nut chewing is a habit only common in Southern Asian nations and has been linked with adverse health consequences, such as oral cancer and mental disorder [30]. In Taiwan, 17.2% of men aged 18 and over in 2007 had the habit of betel-nut chewing [9]. However, the question is that there is insufficient evidence regarding the linkage between betel-nut chewing and health screening participation. Like people with unhealthy behaviors (e.g., tobacco smoking), individuals with the habit of chewing betel nuts could have less perceived need for health screening, because they would tend to ignore health. Therefore, we hypothesize that as an unhealthy behavior, betel-nut chewing is negatively associated with retention in health screening.

Third, current evidence concerning the association between psychiatric disorders and retention in health screening is mixed and controversial. Whereas cognitive impairment has been linked with attrition in health screening [31], some investigators found the impact of psychiatric conditions to be modest at best [32]. Because people with psychiatric problems (e.g., depression) would be more likely to lose interest in seeking health improvement, we hypothesize that psychiatric disorder is associated with a lower rate of retention in health screening.

Fourth, due to the cross-sectional or two-wave short-term design in most previous research, the relationship between retention in long-term health screening has not been well examined and remains debatable, particularly when multiple admission cohorts with varying schedules of follow-ups are involved. We hypothesize that a longer elapsed time between the baseline and follow-up is associated with lower retention. Furthermore, there are significant variations in retention rates across different admission cohorts.

## 2. Materials and Methods

### 2.1. Study Design and Sample Population

Data came from a community-based prospective cohort study “Landseed Integrated Outreaching Neighborhood Screening (LIONS)” which was initiated in 2006 by a regional teaching hospital “Landseed International Hospital” in northern Taiwan to periodically monitor prevailing chronic diseases and related risk factors among adults aged 30 years and over since then. With the support of Taiwan’s health authority for integrated community on-site health screening programs [21], LIONS was implemented in Pingjen, an administrative district of Taoyuan City with over 2 million citizens. A random sample of qualified residents in Pingjen was selected from the household registry stratified by age and gender. They were invited to enroll in the LIONS for free on-site health screening services and an interview with a structured questionnaire [33]. Those screening services include triennial pap smear, biennial mammography, biennial fecal occult blood test, biennial oral mucosal test, blood biomarkers (e.g., serum lipids, fasting glucose), urine tests (e.g., creatinine, albumin), and physical measures (e.g., blood pressure, body mass index, bone mineral density). Furthermore, a questionnaire was administered to collect information on sociodemographic background, history of chronic diseases, medication and dietary habits, health behaviors (e.g., tobacco smoking, alcohol drinking, betel-nut chewing, regular exercise), psychiatric conditions, and health-related quality of life. Participants with abnormal laboratory values at screening were given health education by general physicians during the dissemination of screening reports and advised to seek for medical consultations. Moreover, nurse case managers would monitor high-risk enrollees regularly after the screening.

Due to the data availability, we only selected those LIONS participants enrolled in five annual admission cohorts during 2006–2010 as the study population and then traced those 5901 enrollees till 2014 to evaluate their participation status in three follow-up waves of community health screening (Table 1). All enrollees were contacted two years after the baseline, and two additional follow-ups were made of the five admission cohorts at varying time intervals ranging from one to three years. The varying length of the second and third follow-ups was due to resource and logistical constraints, which may reflect how a community health screening is often implemented in real life by a healthcare organization.

In reference to previous research [34,35], the minimum sample size of a longitudinal study with three repeated binary outcomes (e.g., participation vs. non-participation) could range from 12 to 540 cases (without loss to follow-up) or from 98 to 1107 cases (with the concern of attrition) by using the analytical procedure of generalized estimating equations. Our sample population was greatly larger than above-mentioned minimum numbers, so the findings of this research may be robust from the sample size perspective.

### 2.2. Outcome Variable

Retention was measured by the actual participation in each follow-up health screening (1 = participation, 0 = non-participation). If enrollees did not attend the invited follow-up screening, they would receive a monthly invitation until partaking in a follow-up screening or the end of that year. On average, they would receive up to 3 text messages and 5 phone calls inviting them to attend the follow-up screening. Non-participants included those enrollees who chose not to attend the invited screening of their own volition (e.g., refusal, busyness, and illness), and those who died or dropped out of the LIONS program during the follow-ups.

### 2.3. Covariates

Social determinants included age, gender, and education. Age was classified into three groups (i.e., 30–44, 45–64, and ≥65), and education was measured in years of schooling capped at 17. Health behaviors, including tobacco smoking, alcohol drinking, betel-nut chewing, and regular exercise, were measured as binary variables (1 = yes, 0 = no). Health conditions were indexed by seven dichotomous measures (1 = yes, 0 = no), mainly based on self-reported status (i.e., stroke, cardiac, and hepatic diseases) or combined information on self-reported and laboratory data (i.e., hypertension, type 2 Diabetes mellitus (T2DM), and hyperlipidemia). Finally, the 12-item Chinese Health Questionnaire (CHQ-12) was used to identify psychiatric disorders [36], with a total score of ≥4 indicating at a high risk [37].

To evaluate changes in the odds of retention over time, we calculated the elapsed time as the period (in years) between the baseline enrollment date and the participation date at a given follow-up wave (or the last day of the follow-up year for those non-participants). Moreover, four binary variables were constructed (1 = yes, 0 = no) to indicate memberships of various admission cohorts (with 2006 as the reference).

### 2.4. Statistical Analyses

IBM SPSS 21.0 was employed to implement all analyses in this study. Following the descriptive analysis, generalized estimating equations (GEE) were applied to evaluate retention at each follow-up health screening as a function of social determinants, health conditions, and health behaviors. To ensure a well-defined time sequence, we constructed a time-lagged model in which retention at a given follow-up is a function of covariates measured in the preceding wave.

Arbitrarily ignoring missing data could lead to biased results and reduced precision as well as power [38], especially serious in longitudinal studies [39]. Although the proportions of missing data for most covariates in the GEE model were lower than 5% (Supplementary File Table S1), the pattern of missing data is unlikely to be completely at random, due to the longitudinal design [40]. Therefore, we applied multiple imputations [41] with auxiliary variables [42]. Specifically, five imputed datasets were generated for the GEE analysis. Based on the formula developed by Schafer and Olsen (1998), parameter estimates were computed by averaging across five complete datasets and by adjusting for their variances.

Loss to follow-up (i.e., attrition and death) is a ubiquitous problem in longitudinal studies and can result in serious consequences, such as decreasing sample size, lowering power, and even deteriorating validity [43]. Compared to nonresponse (i.e., missing data), attrition or mortality could have greater impacts on statistical estimates [44], especially when those lost to follow-up people with unique characteristics. Consequently, mortality and attrition (i.e., those enrollees permanently moving out of the Pingjen district or losing the capacity to attend follow-up screenings) were incorporated as confounding variables to adjust for potential selection biases [13].

### 2.5. Ethical Approval

Complying with the Helsinki Declaration and local legislation, all procedures performed in this study were approved by the Institutional Review Board (IRB) of Landseed International Hospital (IRB-16-010-C0). All subjects signed an informed consent form to release their data for this study.

## 3. Results

Table 2 presents the characteristics of sample populations in the three follow-up waves, including retention and other covariates measured at the preceding wave. During the follow-up period, 64 (1.1%) enrollees died, and 188 (3.2%) people left the study, so the sample population declined from 5901 to 5649 persons. Furthermore, the retention rates decreased obviously from 70.7% at the 1st follow-up to 41.7% at the 3rd follow-up.

Of the 5901 enrollees, 45.3% were men, the average schooling year was around 8.3 years, and 86.3% joined the LIONS during 2006–2008. The proportions of enrollees with smoking, drinking, betel-nut chewing, regular exercise, and psychiatric disorders decreased gradually during the follow-ups. In contrast, the prevalence rates of hypertension, T2DM, and hepatic disease increased during 2006–2014. After all, trends of percentages of participants with hyperlipidemia, cardiac disease, and stroke were inconsistent over the follow-up period.

Table 3 exhibits the relationship between retention and various covariates. Compared with middle-aged enrollees, younger adults (with the adjusted odds ratio (aOR) = 0.72, and the 95% confidence interval (CI) from 0.65 to 0.81) and elderly people (aOR = 0.76, 95% CI 0.68–0.86) were less likely to partake in follow-up screenings. Moreover, higher education (aOR = 1.04, 95% CI 1.03–1.05) was related to a greater likelihood of retention.

Due to the arbitrary scaling factor, the magnitude of the odds ratio is difficult to interpret [45]. Hence, we also present the predicted probabilities for retention as a function of various predictors while conditioning all other covariates with their mean values at the baseline (Table 4). Younger and elderly participants had lower probabilities of retention (0.71 and 0.72, respectively), compared with middle-aged enrollees (0.77). College-graduated enrollees had a 4.9% higher retention probability than those with junior high school education.

The linkages between health behaviors and retention appeared to be quite robust (Table 3), even when social determinants and health conditions were controlled. Both tobacco smoking (aOR = 0.75, 95% CI 0.66–0.84) and betel-nut chewing (aOR = 0.70, 95% CI 0.56–0.89) were associated with lower retention; conversely, regular exercise was related to greater likelihood of retention (aOR = 1.20, 95% CI 1.11–1.29). Namely, enrollees with unhealthy behaviors (i.e., tobacco smoking and betel-nut chewing) had lower retention probabilities (0.69 vs. 0.75 and 0.67 vs. 0.75, respectively). In contrast, people with regular exercise had a higher probability of retention (0.76), compared to their sedentary counterparts (0.72).

Chronic conditions were significantly associated with the odds of retention (Table 3). Having hypertension (aOR = 0.85, 95% CI 0.78–0.93), T2DM (aOR = 0.72, 95% CI 0.62–0.82), stroke (aOR = 0.70, 95% CI 0.50–0.98), and psychiatric disorder (aOR = 0.87, 95% CI 0.80–0.94) were correlated with a lower likelihood of retention. Therefore, chronic conditions were related to lower marginal probabilities of retention (Table 4). Specifically, hypertension, T2DM, stroke and psychiatric disorder would reduce the retention probabilities to 0.72, 0.68, 0.67 and 0.72, respectively, from 0.75, 0.75, 0.74, and 0.75.

Table 3 shows that the odds of retention declined substantially as time elapsed (aOR = 0.71, 95% CI 0.70–0.72), and varied significantly across admission cohorts. Relative to the admission cohort of 2006, people enrolled in later cohorts, particularly after 2008, were less likely to be retained (aORs = 0.33–0.83, *p* < 0.01). According to Table 4, a one-year increase in elapsed time reduced the retention probability from 0.80 to 0.73; furthermore, those enrolled after 2006 had significantly lower probabilities of retention. For instance, individuals enrolled in 2009 and 2010 had predicted retention probabilities of 0.62 and 0.55 versus 0.79 for those joining the LIONS in 2006. Finally, mortality (aOR = 0.19, 95% CI 0.12–0.30) and attrition (aOR = 0.24, 95% CI 0.19–0.31) were associated with much lower retention (Table 3), suggesting the importance of controlling them in exploring retention issues.

Based on the predicted probabilities, we could ascertain the relative importance of predictors of retention (Table 4). Variations in admission cohorts led to the greatest reduction in retention probability; particularly, those in the 2009 and 2010 admission cohorts were 17% and 24% less likely to participate in a follow-up screening relative to those in the 2006 admission cohort. In contrast, each of the other significant covariates generally accounted for a 3–7% reduction in the probability of retention.

## 4. Discussion

This research has yielded evidence that prior observations regarding participation in health screening from Western developed nations may apply to Taiwan. Briefly, middle-aged adults and those with higher education were more likely to be retained in health screening. Moreover, those engaged in unhealthy behaviors (i.e., tobacco smoking, betel-nut chewing) and those with psychiatric disorders or chronic conditions (i.e., hypertension, diabetes, and stroke) were less likely to be retained. Finally, retention rates declined significantly with increasing time intervals since enrollment and varied substantially across admission cohorts.

To the best of our knowledge, this research is the first, which analyzes retention in the context of a longitudinal health screening program involving multiple admission cohorts with varying schedules of follow-ups. Prior studies of health screening were largely cross-sectional or longitudinal over a short period of time [46]; thus, there has been a limited understanding of how the probability of retention changes over an extended period of time. According to our findings, retention diminished significantly over time, and there existed significant variations in retention across admission cohorts, even controlling for other important covariates. According to Lugtig’s suggestions in 2014 [47], commitment to the survey, habit, and incentives can positively affect retention, whereas panel fatigue and shocks resulting from life events affect the probability of retention negatively. The substantially lower retention of later admission cohorts (i.e., 2009 and 2010) could be partially attributed to their lower initial interest or commitment to participate. Specifically, those recruited in 2009 and 2010 were first contacted during 2006–2008 and did not want to participate in the health screening but were persuaded later to participate. If the participation at the baseline itself did not change their commitment to the ongoing health screening, these respondents are more likely to drop out in subsequent waves. To enhance retention probabilities, data on the commitment, habit, incentives, panel fatigue, and personal life events of the participants, as well as the non-participants, should be collected. Further research on the patterns of retention over time and their predictors is warranted.

Numerous studies have suggested that relative to middle-aged individuals, younger and older people are less likely to participate in health screening [10,31]. Age differences in health screening participation could be attributed to several factors [15]. It is more difficult to contact young people because they were more likely to relocate or have high mobility due to changed employment, poor financial conditions, unstable relationships, or absence for varying reasons. In contrast, older people are less likely to be retained mainly because of poor health. Nevertheless, there was sparse evidence from non-Western countries regarding the above associations. Our observation of a curvilinear relationship between age and retention in health screening has extended the generalizability of this finding to Taiwan, a non-Western society.

Unhealthy behaviors (e.g., smoking) are linked with lower utilization of preventive services, mainly because of low self-efficacy, low expectation of the efficacy of health checks, and ignoring health [10,15,48]. This study extends our knowledge in this respect by showing a negative relationship between retention and betel-nut chewing in addition to tobacco smoking and alcohol drinking. The present research concludes that such a relationship could be found not only in a cross-sectional [24] but also in a multi-wave longitudinal study of health screening.

There is mixed evidence concerning the relationship between mental disorders and retention, although some investigators have reported that psychiatric disorders were modestly associated with retention because of failure to contact, morbidity, and death [32]. Our finding of an inverse but moderate relationship between psychiatric disorders and retention in Taiwan provides additional support for this proposition.

Surveillance for chronic illnesses is critical to identifying needs and disparities, setting priorities for action, and assessing programmatic progress. A major enhancement of surveillance is longitudinal health screening, which allows a better assessment of both community-based and medical interventions, enabling more sophisticated analyses of what works [49]. The present research illustrates a step in this direction. Meanwhile, longitudinal health screening would facilitate coordination between public health and medical care. Whereas most chronic illnesses are managed in healthcare settings, there is great potential to leverage community-based health screening for both lifestyle interventions and disease management [50,51]. In practice, case managers shall refer those potential patients identified in health screening to appropriate healthcare facilities for seeking professional treatment and continuously monitor the treatment effects. Furthermore, case managers can cooperate with health professionals for delivering integrated preventive care to those people at high risk of certain diseases. Moreover, in implementing longitudinal health screening programs, public health professionals should consider targeting those at higher risk of non-participation as they are precisely those who would significantly benefit from health screening. Finally, given the substantial decline in retention rates over time, greater investment needs to be made to dissemination and implementation research to develop and evaluate various strategies for improving uptake, as well as retention [52].

### Limitations

This study could be improved in several ways. First, although this study was based on a sizable sample with four repeated observations over a 9-year span, data were drawn from one urban district in northern Taiwan, rather than nationally representative data. Further replications with data collected in Taiwan and other Asian countries are warranted for extending the generalizability. Second, we were unable to address the impact of the COVID-19 pandemic in the current study. However, those with chronic diseases have been at significantly higher risk of severe morbidity and mortality due to COVID-19. This may in turn lead to a greater willingness of individuals to seek preventive services, due to increased perceived need. Thus, future research can be directed towards the impacts of the COVID-19 pandemic on changes in health behaviors or health status. Third, self-reported binary measures of health behaviors and health conditions could be further refined by including more information about their gradations, which may reduce measurement errors and improve statistical estimations. Fourth, more information on the reasons for non-participation over time could be useful, despite the adjustment of mortality and attrition in our analysis. Fifth, mixed methods research that integrates quantitative data with qualitative data throughout research endeavors may be warranted [53]. Finally, our research framework based on the Andersen’s behavioral model could be explicitly improved by the inclusion of health literacy. As a mutable factor, health literacy may play an important intermediate role between predisposing variables (i.e., age, gender, education) and perceived need [54,55,56,57]; meanwhile, the modified retention framework becomes more logical and manipulable. Thus, the collection of health literacy data would be necessary.

## 5. Conclusions

In addition to well-documented predictors of health screening, this study also provides some new insights by showing that both betel-nut chewing, like other unhealthy behaviors, and mental disorders are negatively associated with retention. More importantly, greater attention needs to be directed to retention over time and variations across admission cohorts which could have critical implications for the successful implementation of long-term health screening in communities.

## Figures and Tables

**Table 1:** Enrollment and retention situations during 2006–2014 for 5 admission cohorts in the “Landseed Integrated Outreaching Neighborhood Screening (LIONS)” study.

Admission Cohorts	Screening Year								
	2006	2007	2008	2009	2010	2011	2012	2013	2014
2006	1605 *		1137 ^†^			874 ^‡^			649 ^§^
2007		2068 *		1483 ^†^		1155 ^‡^			782 ^§^
2008			1424 *		1066 ^†^	949 ^‡^			654 ^§^
2009				676 *		425 ^†^	321 ^‡^		269 ^§^
2010					128 *		63 ^†^	59 ^‡^	53 ^§^

* Enrollment at baseline (N = 5901, across admission cohorts). ^†^ Retention at 1st follow-up (*n* = 4174, across admission cohorts). ^‡^ Retention at 2nd follow-up (*n* = 3358, across admission cohorts). ^§^ Retention at 3rd follow-up (*n* = 2407, across admission cohorts).

**Table 2:** Descriptive statistics of covariates at three follow-up waves in the LIONS during 2006–2014.

(N_baseline_ = 5901)	1st Follow-Up Wave (N = 5834)	2nd Follow-Up Wave (N = 5778)	3rd Follow-Up Wave (N = 5649)
Covariates *	% or Mean ± SD	% or Mean ± SD	% or Mean ± SD
Retention ^†^	70.7%	57.6%	41.7%
Time since enrollment (years)	2.09 ± 0.32	4.06 ± 0.82	6.96 ± 1.07
Admission cohorts			
2006	27.2%		
2007	35.0%		
2008	24.1%		
2009	11.5%		
2010	2.2%		
Gender (male)	45.4%		
Age	53.42 ± 13.23	54.84 ± 13.19	56.02 ± 13.18
30–44	26.6%	22.8%	20.3%
45–64	53.4%	55.2%	56.0%
≥65	20.0%	22.0%	23.6%
Education (years)	8.25 ± 4.65		
Tobacco smoking (yes)	17.7%	16.0%	14.8%
Alcohol drinking (yes)	12.3%	9.4%	7.7%
Betel-nut chewing (yes)	3.1%	2.6%	2.4%
Regular exercise (yes)	64.1%	63.7%	61.0%
Psychiatric disorder (yes)	27.1%	24.4%	22.3%
Hypertension (yes)	32.0%	33.3%	36.4%
T2DM (yes)	8.9%	9.8%	10.9%
Hyperlipidemia (yes)	16.8%	14.6%	14.7%
Cardiac disease (yes)	5.9%	5.5%	5.9%
Stroke (yes)	1.3%	1.2%	1.2%
Hepatic disease (yes)	8.3%	9.4%	9.5%
Lost to follow-up ^‡^	67 (1.1%)	123 (2.1%)	252 (4.3%)
Mortality	20 (0.3%)	33 (0.6%)	64 (1.1%)
Attrition	47 (0.8%)	90 (1.5%)	188 (3.2%)

* All predictors were measured at the preceding wave with participation, except for retention (non-predictor), time since enrollment, admission cohorts (at the baseline), and lost to follow-up. For example, predictors in the 1st follow-up wave had the values measured at the enrollment (i.e., baseline wave). ^†^ Retention was measured at the follow-up wave as: N_(participants at n follow-up)_/N_(sample at n − 1 follow-up)._ ^‡^ Subjects died (i.e., mortality) or dropped out the LIONS (i.e., attrition) after the enrollment. Abbreviation: T2DM, type 2 Diabetes mellitus; SD, standard deviation.

**Table 3:** GEE ^†^ logistic regression model of retention in the LIONS during 2006–2014.

Covariates	Full Model ^‡^
	Exp(*β*) (95% CI)
Intercept	7.35 (6.26, 8.63) ***
Time since enrollment (years)	0.71 (0.70, 0.72) ***
Admission cohorts (ref. = 2006)	
2007	0.78 (0.70, 0.87) ***
2008	0.83 (0.73, 0.93) **
2009	0.43 (0.37, 0.50) ***
2010	0.33 (0.24, 0.44) ***
Mortality (yes = 1)	0.19 (0.12, 0.30) ***
Attrition (yes = 1)	0.24 (0.19, 0.31) ***
Gender (male = 1)	0.94 (0.85, 1.04)
Age (ref. = 45–64)	
30–44	0.72 (0.65, 0.81) ***
≥65	0.76 (0.68, 0.86) ***
Education (years)	1.04 (1.03, 1.05) ***
Tobacco smoking (yes = 1)	0.75 (0.66, 0.84) ***
Alcohol drinking (yes = 1)	1.00 (0.88, 1.13)
Betel-nut chewing (yes = 1)	0.70 (0.56, 0.89) **
Regular exercise (yes = 1)	1.20 (1.11, 1.29) ***
Psychiatric disorder (yes = 1)	0.87 (0.80, 0.94) ***
Hypertension (yes = 1)	0.85 (0.78, 0.93) ***
T2DM (yes = 1)	0.72 (0.62, 0.82) ***
Hyperlipidemia (yes = 1)	1.08 (0.98, 1.19)
Cardiac disease (yes = 1)	1.05 (0.89, 1.23)
Stroke (yes = 1)	0.70 (0.50, 0.98) *
Hepatic disease (yes = 1)	1.00 (0.88, 1.14)

^†^ GEE model with “probability of non-participation” as the reference group. ^‡^ Adjusted odds ratio (95% CI) by all other covariates in the model (detailed subgroup information, such as sample size or mean, on each covariate with reference to Table 2). * *p* < 0.05, ** *p* < 0.01, *** *p* < 0.001. Abbreviation: GEE, generalized estimating equation; CI, confidence interval; T2DM, type 2 Diabetes mellitus.

**Table 4:** Predicted probabilities of retention in the LIONS during 2006–2014.

Covariates	Conditions ^‡^ (Means or %)	Predicted Probability ^†,§^
Time since enrollment (years)	2.088	
1 year		0.795
2 years		0.733
Admission cohorts		
2006 (ref.)	27.2%	0.788
2007	35.0%	0.745
2008	24.1%	0.755
2009	11.5%	0.617
2010	2.2%	0.550
AGE		
45-64 (ref.)	53.4%	0.769
30–44	26.6%	0.707
≥65	20.0%	0.717
Education (years)	8.251	
9 years		0.749
16 years		0.798
Tobacco smoking		
No (ref.)		0.753
Yes	17.7%	0.694
Betel-nut chewing		
No (ref.)		0.745
Yes	3.1%	0.672
Regular exercise		
No (ref.)		0.720
Yes	64.1%	0.755
Psychiatric disorder		
No (ref.)		0.750
Yes	27.1%	0.723
Hypertension		
No (ref.)		0.753
Yes	32.0%	0.721
T2DM		
No (ref.)		0.749
Yes	8.9%	0.678
Stroke		
No (ref.)		0.744
Yes	1.3%	0.671
Dead (yes)	1.1%	
Drop out (yes)	3.2%	
Gender (male)	45.4%	
Alcohol drinking (yes)	12.3%	
Hyperlipidemia (yes)	16.8%	
Cardiac disease (yes)	5.9%	
Hepatic disease (yes)	8.3%	

^†^ Probabilities were derived from the GEE model in Table 3 with covariates conditioned at the baseline average values (means or %). Thus, the logistic equation of GEE model could be modified as: *f*(*x*) = Ln [Probability/(1 − Probability)] = 1.995 + (−0.346) × Time + (−0.245) × dummy_2007 + (−0.191) × dummy_2008 + (−0.840) × dummy_2009 + (−1.115) × dummy_2010 + (−0.323) × dummy_AGE_30–44_ + (−0.272) × dummy_AGE_65_ + 0.040 × Education + (−0.295) × dummy_Smoking + (−0.355) × dummy_Chewing_betel-nut_ + 0.182 × dummy_Exercise + (−0.138) × dummy_Psychiatric + (−0.162) × dummy_Hypertension + (−0.336) × dummy_T2DM + (−0.356) × dummy_Stroke + (−1.652) × 0.011 + (−1.432) × 0.032 + (−0.060) × 0.454 + (−0.003) × 0.123 + 0.073 × 0.168 + 0.044 × 0.059 + (−0.003) × 0.083; ^‡^ Conditions were the mean or percentage values of covariates at the baseline. In addition, the conditions of significant covariates in the GEE model were used to replace the dummy values when those covariates were not under the prediction. ^§^ The predicted probability of retention for a certain covariate by conditioning other covariates at the baseline average values was calculated as: Predicted probability $=\frac{e^{f(x)}}{1+e^{f(x)}}$ Abbreviation: T2DM, type 2 Diabetes mellitus.

## Data Availability

Data sharing is not applicable to this article.

## Supplementary Materials

The following supporting information can be downloaded at: https://www.mdpi.com/article/10.3390/ijerph19116813/s1, Table S1: Missing data of covariates at three follow-up waves in the “Landseed Integrated Outreaching Neighborhood Screening (LIONS)” study, Taiwan 2006–2014.
