# Supplementary material for: Retention in Community Health Screening among Taiwanese Adults: A 9-Year Prospective Cohort Study
Source: Int J Environ Res Public Health. 2022 Jun 2;19(11):6813. doi: 10.3390/ijerph19116813 (PMC9180367; doi:10.3390/ijerph19116813)
Supplement: Supplementary file 1 [file ijerph-19-06813-s001.zip › Supplementary File S4 -- GEE syntax.pdf]

************************** full GEE model with the control of Mortality and Attrition ********************************

```
GENLIN Participation (REFERENCE=FIRST) BY Gender Smoking Drinking Chewing Exercise Mental
    Hypertension Hyperglycemia CHO_G2 Cardiac Stroke Hepatic Cohorts Died Drop_out AGE_G3T1
    (ORDER=DESCENDING) WITH Time Edu_Y_Medium
  /MODEL Time Gender Edu_Y_Medium Smoking Drinking Chewing Exercise Mental Hypertension
    Hyperglycemia CHO_G2 Cardiac Stroke Hepatic Cohorts Died Drop_out AGE_G3T1 INTERCEPT=YES
 DISTRIBUTION=BINOMIAL LINK=LOGIT
  /CRITERIA METHOD=FISHER(1) SCALE=PEARSON MAXITERATIONS=100 MAXSTEPHALVING=5
    PCONVERGE=1E-006(ABSOLUTE) SINGULAR=1E-012 ANALYSISTYPE=3(WALD) CILEVEL=95 LIKELIHOOD=FULL
  /EMMEANS SCALE=ORIGINAL
  /EMMEANS TABLES=Gender SCALE=ORIGINAL COMPARE=Gender PADJUST=LSD
  /EMMEANS TABLES=Smoking SCALE=ORIGINAL COMPARE=Smoking PADJUST=LSD
  /EMMEANS TABLES=Drinking SCALE=ORIGINAL COMPARE=Drinking PADJUST=LSD
  /EMMEANS TABLES=Chewing SCALE=ORIGINAL COMPARE=Chewing PADJUST=LSD
  /EMMEANS TABLES=Exercise SCALE=ORIGINAL COMPARE=Exercise PADJUST=LSD
  /EMMEANS TABLES=Mental SCALE=ORIGINAL COMPARE=Mental PADJUST=LSD
  /EMMEANS TABLES=Hypertension SCALE=ORIGINAL COMPARE=Hypertension PADJUST=LSD
  /EMMEANS TABLES=Hyperglycemia SCALE=ORIGINAL COMPARE=Hyperglycemia PADJUST=LSD
  /EMMEANS TABLES=CHO_G2 SCALE=ORIGINAL COMPARE=CHO_G2 PADJUST=LSD
  /EMMEANS TABLES=Cardiac SCALE=ORIGINAL COMPARE=Cardiac PADJUST=LSD
  /EMMEANS TABLES=Stroke SCALE=ORIGINAL COMPARE=Stroke PADJUST=LSD
  /EMMEANS TABLES=Hepatic SCALE=ORIGINAL COMPARE=Hepatic PADJUST=LSD
  /EMMEANS TABLES=Cohorts SCALE=ORIGINAL COMPARE=Cohorts PADJUST=LSD
  /EMMEANS TABLES=Died SCALE=ORIGINAL COMPARE=Died PADJUST=LSD
  /EMMEANS TABLES=Drop_out SCALE=ORIGINAL COMPARE=Drop_out PADJUST=LSD
```

```
   /EMMEANS TABLES=AGE_G3T1 SCALE=ORIGINAL COMPARE=AGE_G3T1 PADJUST=LSD
   /REPEATED SUBJECT=ID SORT=YES CORRTYPE=AR(1) ADJUSTCORR=YES COVB=ROBUST MAXITERATIONS=100
      PCONVERGE=1e-006(ABSOLUTE) UPDATECORR=1
   /MISSING CLASSMISSING=EXCLUDE
   /PRINT CPS DESCRIPTIVES MODELINFO FIT SUMMARY SOLUTION (EXPONENTIATED).
```

```
************************** full GEE model without controlling Mortality and Attrition ********************************

GENLIN Participation (REFERENCE=FIRST) BY Gender Smoking Drinking Chewing Exercise Mental
      Hypertension Hyperglycemia CHO_G2 Cardiac Stroke Hepatic Cohorts AGE_G3T1
      (ORDER=DESCENDING) WITH Time Edu_Y_Medium
   /MODEL Time Gender Edu_Y_Medium Smoking Drinking Chewing Exercise Mental Hypertension
      Hyperglycemia CHO_G2 Cardiac Stroke Hepatic Cohorts AGE_G3T1 INTERCEPT=YES
  DISTRIBUTION=BINOMIAL LINK=LOGIT
   /CRITERIA METHOD=FISHER(1) SCALE=PEARSON MAXITERATIONS=100 MAXSTEPHALVING=5
      PCONVERGE=1E-006(ABSOLUTE) SINGULAR=1E-012 ANALYSISTYPE=3(WALD) CILEVEL=95 LIKELIHOOD=FULL
   /EMMEANS SCALE=ORIGINAL
   /EMMEANS TABLES=Gender SCALE=ORIGINAL COMPARE=Gender PADJUST=LSD
   /EMMEANS TABLES=Smoking SCALE=ORIGINAL COMPARE=Smoking PADJUST=LSD
   /EMMEANS TABLES=Drinking SCALE=ORIGINAL COMPARE=Drinking PADJUST=LSD
   /EMMEANS TABLES=Chewing SCALE=ORIGINAL COMPARE=Chewing PADJUST=LSD
   /EMMEANS TABLES=Exercise SCALE=ORIGINAL COMPARE=Exercise PADJUST=LSD
```

```
/EMMEANS TABLES=Mental SCALE=ORIGINAL COMPARE=Mental PADJUST=LSD
/EMMEANS TABLES=Hypertension SCALE=ORIGINAL COMPARE=Hypertension PADJUST=LSD
/EMMEANS TABLES=Hyperglycemia SCALE=ORIGINAL COMPARE=Hyperglycemia PADJUST=LSD
/EMMEANS TABLES=CHO_G2 SCALE=ORIGINAL COMPARE=CHO_G2 PADJUST=LSD
/EMMEANS TABLES=Cardiac SCALE=ORIGINAL COMPARE=Cardiac PADJUST=LSD
/EMMEANS TABLES=Stroke SCALE=ORIGINAL COMPARE=Stroke PADJUST=LSD
/EMMEANS TABLES=Hepatic SCALE=ORIGINAL COMPARE=Hepatic PADJUST=LSD
/EMMEANS TABLES=Cohorts SCALE=ORIGINAL COMPARE=Cohorts PADJUST=LSD
/EMMEANS TABLES=AGE_G3T1 SCALE=ORIGINAL COMPARE=AGE_G3T1 PADJUST=LSD
/REPEATED SUBJECT=ID SORT=YES CORRTYPE=AR(1) ADJUSTCORR=YES COVB=ROBUST MAXITERATIONS=100
  PCONVERGE=1e-006(ABSOLUTE) UPDATECORR=1
/MISSING CLASSMISSING=EXCLUDE
/PRINT CPS DESCRIPTIVES MODELINFO FIT SUMMARY SOLUTION (EXPONENTIATED).
```
