# Supplementary material for: Retention in Community Health Screening among Taiwanese Adults: A 9-Year Prospective Cohort Study
Source: Int J Environ Res Public Health. 2022 Jun 2;19(11):6813. doi: 10.3390/ijerph19116813 (PMC9180367; doi:10.3390/ijerph19116813)
Supplement: Supplementary file 1 [file ijerph-19-06813-s001.zip › Supplementary File Table S1.pdf]

Supplementary File Table S1. Missing data of covariates at three follow-up waves† in the "Landseed Integrated Outreaching Neighborhood Screening (LIONS)" study, Taiwan 2006-2014

($N_{initial}$=5,901)	1st follow-up (N=5,834)	2nd follow-up (N=5,778)	3rd follow-up (N=5,649)
Covariates†	n(%)	n(%)	n(%)
Gender	0(0)	0(0)	0(0)
Age	0(0)	0(0)	0(0)
Education	79(1.4)	77(1.3)	76(1.3)
Tobacco smoking	241(4.1)	117(2.0)	111(2.0)
Alcohol drinking	265(4.5)	182(3.1)	156(2.8)
Betel-nut chewing	252(4.3)	124(2.1)	110(1.9)
Regular exercise	280(4.8)	196(3.4)	133(2.4)
Psychiatric disorder	345(5.9)	272(4.7)	208(3.7)
Hypertension	0(0)	0(0)	0(0)
T2DM	0(0)	0(0)	0(0)
Hyperlipidemia	8(0.1)	5(0.1)	6(0.1)
Cardiac disease	429(7.4)	287(5.0)	178(3.2)
Stroke	327(5.6)	222(3.8)	146(2.6)
Hepatic disease	666(11.4)	366(6.3)	240(4.2)
Lost to follow-up‡			
Mortality	20	33	64
Attrition	47	90	188
Household income	771(13.2)	727(12.6)	566(10.0)
Marital status	268(4.6)	152(2.6)	140(2.5)

†All covariates were measured at the preceding wave with participation, except for lost to follow-up. ‡Subjects died (i.e., mortality) or dropped out the LIONS (i.e., attrition) after the enrollment. *Abbreviation*: T2DM, type 2 Diabetes mellitus.
