# Supplementary material for: Retention in Community Health Screening among Taiwanese Adults: A 9-Year Prospective Cohort Study
Source: Int J Environ Res Public Health. 2022 Jun 2;19(11):6813. doi: 10.3390/ijerph19116813 (PMC9180367; doi:10.3390/ijerph19116813)
Supplement: Supplementary file 1 [file ijerph-19-06813-s001.zip › Supplementary File Table S2.pdf]

Supplementary File Table S2. The comparison among the coefficients of GEE† models with or without multiple imputation or control of attrition and mortality

Covariates	Model 1‡	Model 2‡	Model 3‡	Model 4‡
Intercept	1.995***	2.226***	1.872***	2.105***
Time since enrollment (years)	-0.346***	-0.352***	-0.338***	-0.345***
Admission cohorts (ref. = 2006)				
2007	-0.245***	-0.365***	-0.238***	-0.362***
2008	-0.191**	-0.343***	-0.171**	-0.327***
2009	-0.840***	-0.972***	-0.820***	-0.959***
2010	-1.115***	-1.285***	-1.093***	-1.263***
Mortality (yes = 1)	-1.652***	-1.770***		
Attrition (yes = 1)	-1.432***	-1.393***		
Gender (male = 1)	-0.060	-0.032	-0.073	-0.037
Age (ref. = 45-64)				
30-44	-0.323***	-0.300***	-0.330***	-0.298***
≧65	-0.272***	-0.246***	-0.254***	-0.233***
Education (years)	0.040***	0.035***	0.043***	0.037***
Tobacco smoking (yes = 1)	-0.295***	-0.342***	-0.279***	-0.334***
Alcohol drinking (yes = 1)	-0.003	-0.047	0.010	-0.037
Betel-nut chewing (yes = 1)	-0.355**	-0.395**	-0.335**	-0.390**
Regular exercise (yes = 1)	0.182***	0.191***	0.176***	0.189***
Psychiatric disorder (yes = 1)	-0.138***	-0.105*	-0.134***	-0.097*
Hypertension (yes = 1)	-0.162***	-0.169***	-0.167***	-0.171***
T2DM (yes = 1)	-0.336***	-0.328***	-0.336***	-0.325***
Hyperlipidemia (yes = 1)	0.073	0.074	0.072	0.072
Cardiac disease (yes = 1)	0.044	0.011	0.039	0.007
Stroke (yes = 1)	-0.356*	-0.411*	-0.325	-0.381*
Hepatic disease (yes = 1)	-0.003	0.091	-0.013	0.090

†GEE model with "probability of non-participation" as the reference group. ‡Model 1 with multiple imputation and control of attrition and mortality, Model 2 without multiple imputation but with control of attrition and mortality, Model 3 with multiple imputation but without control of attrition and mortality, and Model 4 without multiple imputation and control of attrition and mortality. * $p < 0.05$, ** $p < 0.01$, *** $p < 0.001$. *Abbreviation*: GEE, generalized estimating equation; T2DM, type 2 Diabetes mellitus.
