# Supplementary material for: Retention in Community Health Screening among Taiwanese Adults: A 9-Year Prospective Cohort Study
Source: Int J Environ Res Public Health. 2022 Jun 2;19(11):6813. doi: 10.3390/ijerph19116813 (PMC9180367; doi:10.3390/ijerph19116813)
Supplement: Supplementary file 1 [file ijerph-19-06813-s001.zip › Supplementary File Table S3.pdf]

Supplementary File Table S3. The comparison of covariates at baseline between retention and loss to follow-up subgroups of the LIONS enrollees in Taiwan during 2006-2014

($N_{initial}$=5,901)	Retention (N=5,649)	Loss to follow-up[†] (N=252)	*p*-value
Covariates	Mean±SD or n(%)	Mean±SD or n(%)	
Gender			
Female	3,090(54.7)	132(52.4)	0.469
Male	2,559(45.3)	120(47.6)	
Age			
30-44	1,500(26.6)	68(27.0)	0.699
45-64	3,025(53.5)	129(51.2)	
≧65	1,124(19.9)	55(21.8)	
Education (years)	8.31±4.64	7.52±4.80	0.011
No schooling	459(8.2)	31(13.0)	0.034
below Senior high school	2,173(39.0)	98(41.2)	
Senior high school	1,709(30.7)	65(27.3)	
Undergraduate or above	1,232(22.1)	44(18.5)	
Marital status			
Never married	266(4.9)	10(4.3)	0.631
Married & living together	4,795(89.0)	210(90.9)	
Others	329(6.1)	11(4.8)	
Household income (yearly)[‡]			
＜NT$300,000	3,095(63.1)	134(63.8)	0.733
NT$300,000~750,000	1,332(27.2)	53(25.2)	
＞NT$750,000	475(9.7)	23(11.0)	
Tobacco smoking			
Never or ceased	4,436(81.9)	190(81.5)	0.889
Continuously	980(18.1)	43(18.5)	
Alcohol drinking			
Never or ceased	4,708(87.3)	203(87.5)	0.928
Continuously	685(12.7)	29(12.5)	
Betel-nut chewing			
Never or ceased	5,234(96.8)	224(96.1)	0.565
Continuously	171(3.2)	9(3.9)	
Regular exercise			

No	1,921(35.7)	88(38.4)	0.400
Yes	3,459(64.3)	141(61.6)	
BMI	24.50±3.62	24.79±3.78	0.212
＜24.0	2,664(47.4)	110(44.0)	0.295
≧24.0	2,959(52.6)	140(56.0)	
Psychiatric disorder			
No	3,908(73.5)	162(71.7)	0.544
Yes	1,409(26.5)	64(28.3)	
Hypertension			
No	3,553(65.8)	138(58.7)	0.026
Yes	1,847(34.2)	97(41.3)	
Diabetes mellitus (type 2)			
No	4,746(89.9)	198(87.2)	0.192
Yes	533(10.1)	29(12.8)	
Hyperlipidemia			
No	3,245(60.7)	127(54.3)	0.051
Yes	2,105(39.3)	107(45.7)	
Cardiac disease			
No	4,946(94.4)	206(92.4)	0.190
Yes	291(5.6)	17(7.6)	
Stroke			
No	5,272(98.8)	227(99.1)	0.506
Yes	62(1.2)	2(0.9)	
Hepatic disease			
No	4,645(92.8)	191(91.4)	0.458
Yes	363(7.2)	18(8.6)	

†Subjects died (i.e., mortality) or dropped out the LIONS (i.e., attrition) after the enrollment. ‡NT$ means New Taiwan dollar; currently, 1 US dollar = 29.5 NT dollars.
